# Development of a Minimally Invasive Screening Tool to Identify Obese Pediatric Population at Risk of Obstructive Sleep Apnea/Hypopnea Syndrome

**DOI:** 10.3390/bioengineering7040131

**Published:** 2020-10-19

**Authors:** José Miguel Calderón, Julio Álvarez-Pitti, Irene Cuenca, Francisco Ponce, Pau Redon

**Affiliations:** 1 Fundación Investigación Hospital Clínico (INCLIVA), Avda. Menedez Pelayo 4, 46010 Valencia, Spain; jocalte85@gmail.com (J.M.C.); irene.cuenca95@gmail.com (I.C.); 2 Pediatric Department, Consorcio Hospital General Universitario de Valencia, Avda. Tres Cruces s/n, 46014 Valencia, Spain; japnago@gmail.com (J.Á.-P.); siscopz80@gmail.com (F.P.); 3 CIBEROBN, Health Institute Carlos III, Av. Monforte de Lemos, 3-5. Pavilion 11, 28029 Madrid, Spain

**Keywords:** machine learning, oxygen saturation signal, obstructive sleep apnea syndrome, obese pediatric population

## Abstract

Obstructive sleep apnea syndrome is a reduction of the airflow during sleep which not only produces a reduction in sleep quality but also has major health consequences. The prevalence in the obese pediatric population can surpass 50%, and polysomnography is the current gold standard method for its diagnosis. Unfortunately, it is expensive, disturbing and time-consuming for experienced professionals. The objective is to develop a patient-friendly screening tool for the obese pediatric population to identify those children at higher risk of suffering from this syndrome. Three supervised learning classifier algorithms (i.e., logistic regression, support vector machine and AdaBoost) common in the field of machine learning were trained and tested on two very different datasets where oxygen saturation raw signal was recorded. The first dataset was the Childhood Adenotonsillectomy Trial (CHAT) consisting of 453 individuals, with ages between 5 and 9 years old and one-third of the patients being obese. Cross-validation was performed on the second dataset from an obesity assessment consult at the Pediatric Department of the Hospital General Universitario of Valencia. A total of 27 patients were recruited between 5 and 17 years old; 42% were girls and 63% were obese. The performance of each algorithm was evaluated based on key performance indicators (e.g., area under the curve, accuracy, recall, specificity and positive predicted value). The logistic regression algorithm outperformed (accuracy = 0.79, specificity = 0.96, area under the curve = 0.9, recall = 0.62 and positive predictive value = 0.94) the support vector machine and the AdaBoost algorithm when trained with the CHAT datasets. Cross-validation tests, using the Hospital General de Valencia (HG) dataset, confirmed the higher performance of the logistic regression algorithm in comparison with the others. In addition, only a minor loss of performance (accuracy = 0.75, specificity = 0.88, area under the curve = 0.85, recall = 0.62 and positive predictive value = 0.83) was observed despite the differences between the datasets. The proposed minimally invasive screening tool has shown promising performance when it comes to identifying children at risk of suffering obstructive sleep apnea syndrome. Moreover, it is ideal to be implemented in an outpatient consult in primary and secondary care.

## 1. Introduction

The obstructive sleep apnea syndrome (OSAS) is a health problem characterized by recurrent episodes of reduction of airflow. These can be partial (hypopnea) or complete (apnea). According to the American Academy of Sleep Medicine (AASM), apnea is defined as an airflow reduction of at least 90% lasting for at least 10 s, while a hypopnea is a 30% reduction for at least 10 s or at least a 3% reduction in oxygen saturation (SpO2) in comparison with the pre-event baseline or associated with arousal [1,2]. The frequency of these events in a fixed time interval, 1 h, is called the apnea/hypopnea index (AHI) and is used to classify the severity of the OSAS syndrome into three levels: mild (5 ≤ AHI < 15), moderate (15 ≤ AHI < 30) and severe (30 ≤ AHI) [1,2]. OSAS can cause a deterioration of the sleep quality as well as other major negative consequences (e.g., behavior regulation, compliance, aggression, impulsivity, hyperactivity, anxiety, depressed mood, emotion regulation and neurocognitive deficits) [3,4]. Its prevalence in children ranges between 0.2% and 4%, increasing in the obese population up to over 50% [3].

Full night polysomnography (PSG) has been recognized by numerous professionals as the gold standard procedure to diagnose OSAS in children and adults [5,6,7,8]. Generally speaking, it consists of a minimum of 11 channels recording different signals like the electroencephalogram (EEG), electrooculogram (EOG), electromyogram (EMG) and electrocardiogram (ECG). Unfortunately, despite the accuracy of the results, it is very disturbing for the patient, particularly in the pediatric population, and expensive because it requires special infrastructure and trained personnel. In an attempt to overcome these limitations, other methods have been proposed (e.g., respiratory polygraphy (RP), pediatric sleep questionnaire (PSQ), sleep clinical record (SCR), nocturnal oximetry studies (NOS)) [9].

Out of all the listed alternative methods, the authors are especially interested in those which can be performed in an outpatient context. This is especially relevant for screening and follow-up strategies in the obese pediatric population. The first of them is the RP, which reduces the invasiveness of the procedure by reducing the number of signals being recorded. Usually, the oronasal flow, chest and abdomen movements, heart rate and blood oxygen are the key parameters measured. Different tests have demonstrated that RP and home-based RP (HRP) are suitable for diagnosing OSAS in children and adolescents [10,11]. The second is the PSQ test. It consists of a set of questions related to 22 symptom items that ask about snoring frequency, loud snoring, observed apneas, difficulty breathing during sleep, daytime sleepiness, inattentive or hyperactive behavior and other pediatric OSAS features. It is oriented to children aged between 2 and 18 years old, and the sleep-related breathing disorder (SRBD) scale can predict the risk to an extent useful for research but not reliable enough for most individual patients [12,13]. The third is the NOS procedure, which measures the arterial oxygen saturation by the detection of pulsatile blood flow. The performance of continuous oximetry overnight can help to identify desaturation patterns useful to identify OSAS in children. As pointed out by Singh et al. [14], this method has been extensively used in the field of sleep medicine and has been classified as a type 4 monitoring device. It is cheap, available and easy to perform on an outpatient basis.

Despite the evident differences between all of these methods, they still require trained personnel to interpret the results. In some cases, this can be time-consuming even if a signal processing software is available with the hardware. Besides the increased physician workload that can be associated with such testing, the results are strongly dependent on the experience of the operator, and strong variabilities in the diagnosis might occur between experts.

In the past two decades, multiple solutions have been proposed to reduce or mitigate such dependencies in adults and pediatric populations (see Table 1). Generally speaking, these are characterized by a set of features extracted from one or several recorded biosignals and a classifier for automatic diagnosis of OSAS severity (see Table 1). Mostafa et al. [15] recently reviewed the solutions available for the adult population. Initially, univariate analyses were employed, and now machine learning (ML) and deep learning (DL) algorithms are proliferating. These can either identify events directly from a register or based on a global analysis of the data.

For the pediatric population, there seem to coexist ML- and DL-based solutions with similar performance between them. The threshold values used to classify severity have a greater impact on performance in this cohort than in adults.

Table 1 presents a summary of the performance of each these tools, including the results regarding sensitivity, specificity and accuracy of solutions focusing on the SpO2 signal. Even though the differences in datasets, sample sizes and methods do not allow for direct comparison between them, this information is useful to benchmark the performance of new tools. Accuracy is similar between both populations, while greater specificity and sensitivity are observed in the adult population.

Unfortunately, the development of this type of solution is not accessible to everyone and in some cases might require a continuous update. In this context, the main objective of this paper is to develop a patient-friendly screening tool oriented to the pediatric population and feasible to be implemented on an outpatient basis. Moreover, this solution should be easy to run, minimizing the interaction of the physicians throughout the whole process and keeping them updated without the need for domain-specific skills. This will allow the popularizing of this type of diagnostic test, which can be especially relevant for specific pediatric cohorts like the obese population.

## 2. Materials and Methods

### 2.1. Datasets

Two datasets were used in this study. The first one was the Childhood Adenotonsillectomy Trial (CHAT), obtained from National Sleep Research Resource (NSRR), [6,35,36,37] with 453 children with ages ranging between 5 and 10 years old. Fifty-two percent of the registries corresponded to girls, one-third of the population was obese (defined as BMI percentile at or above 95%) and the predominant race was black (55%). All participants underwent polysomnography in the sleep laboratory of the hospital in which the minimum period of lights off was 7 h. A total of 43% of them suffered severe OSAS.

Out of all the variables available from this dataset, only those related to oxygen saturation (SpO2) were considered in the present study. The main reason is that apneas and hypopneas are both defined by this parameter. This signal was recorded in the CHAT with a pulse-oximeter, Nonin Model 8000J or comparable, at a sampling rate of 10 Hz or higher. The extracted features from the SpO2 signal are listed and defined in Table 2 [6,35,36,37]. It is worth noting that the oxygen desaturation index (ODI) has been considered as the hourly average number of desaturation episodes, defined as at events lasting >10 s with at least a 3% decrease in saturation from the average saturation in the preceding 120 s [18]. Based on the ODI, the desaturation periods were tagged as hypopnea or apneas. All subjects were classified according to AHI (ahi_a0h3a). Patients with an AHI < 5 were labeled as healthy, whereas patients with an AHI ≥ 5 were considered at risk of suffering OSAS syndrome.

The second dataset consisted of 27 subjects, between 5 and 17 years of age and recruited from those attending an obesity assessment consult at the Pediatric Department of Hospital General of Valencia. All were Caucasian, 42% were girls and 63% were obese (defined as BMI percentile at or above 95%). All participants underwent an HRP using the Philips Alice Respironics device with a minimum of 6 h of time in bed (TIB) and 3 h of sleep time [38]. The recorded signals were airflow, thoracic and abdominal movement, SpO2, body position, ECG and snore. Only the SpO2 raw signal was processed to extract features and calculate the variables of interest according to the definitions shown in Table 2. To do so, MATLAB software was employed.

Finally, to allow direct comparison of the variables from both datasets, a standardization process was performed. This consisted of calculating the Z-score value for each of them.

### 2.2. Machine Learning Algorithms

Three popular ML algorithms specially oriented to supervised learning were used in this study. The first of them is the logistic regression (LR) model, which uses a weighted least square algorithm to predict the regression line that best fits the data points by minimizing the weighted sum of the square distances to the fitted regression line. It is simple and easy to implement and can relate one dependent variable with one or several independent variables. The second is the support vector machine (SVM), which tries to model the input variables by finding the separating boundary (i.e., hyperplane) to reach the classification of the input variables [39]. The third is the AdaBoost (AB) model, which is a common ensemble method that combines in series multiple weak classifiers to generate a strong one. These algorithms from the Scikit-learn library [40] were trained and afterward tested in terms of their capability to classify the subjects. Each classification algorithm has its own set of parameters that can be modified to obtain a better performance. SVM was implemented with an ‘rbg’ kernel, a penalty parameter C from 0.01 to 100 and a gamma parameter from 0.001 to 100. LR classifier was run with L1 penalty and a C, inverse of regularization strength, from 0.01 to 100. AdaBoost was implemented with Decision Tree as a base estimator, a learning rate parameter from 0.01 to 1 and several estimators from 50 to 1000 with an interval of 10. The machine learning algorithms were implemented in Python [41,42].

The CHAT dataset was used to train and test each of the classifiers according to the extracted features from the SpO2 raw signal. A 15 k-fold approach was used. Balanced datasets were used. On the other hand, cross-validation was performed using the Hospital General de Valencia (HG) dataset, which is only composed of an obese population ranging from 9 to 17 years of age. The same features as those in the CHAT dataset were calculated from the raw SpO2 signal using MATLAB software.

Balanced datasets were used to evaluate the performance of each model based on the following parameters: recall or sensitivity, precision or positive predictive value (PPV), specificity, negative predictive value (NPV), accuracy, F1 score and area under the curve (AUC). The recall or sensitivity and precision or PPV are two very important parameters for assessing the performance in identifying unhealthy patients (i.e., denoted as positive subjects in the current manuscript). The former is the portion of real positive cases that are correctly predicted positive, while the latter denotes the portion of predicted positive cases that are really positive. Mathematically, they are defined according to Equations (1) and (2), respectively.

Sensitivity = True Positive/(True Positive + False Negative)(1)

PPV = True Positive/(True Positive + False Positive)(2)

The specificity and the negative predictive value (NPV) are respectively homologous to the two previously described parameters but assess the performance of the model regarding its capability to identify the negative values or healthy subjects. They are mathematically defined by Equations (3) and (4).

Specificity = True Negative/(True Negative + False Positive)(3)

NPV = True Negative/(True Negative + False Negative)(4)

Accuracy and F1 score are two parameters frequently used to assess overall performance. Accuracy is the portion of correctly identified cases, independently of being positive or negative, from the total number of samples (see Equation (5)). The F1 score is the harmonic mean between PPV and sensitivity. Out of the three different types of averages that can be calculated (i.e., arithmetic, geometric and harmonic), the harmonic average is the most conservative of them all; in other words, it is the one that yields the lowest value. It is defined by Equation (6).

Accuracy = (True Positive + True Negative)/(Sample size)(5)

F1 score = 2 × True Positive/(2 × True Positive + False Positive + False Negative)(6)

**Figure 1:**
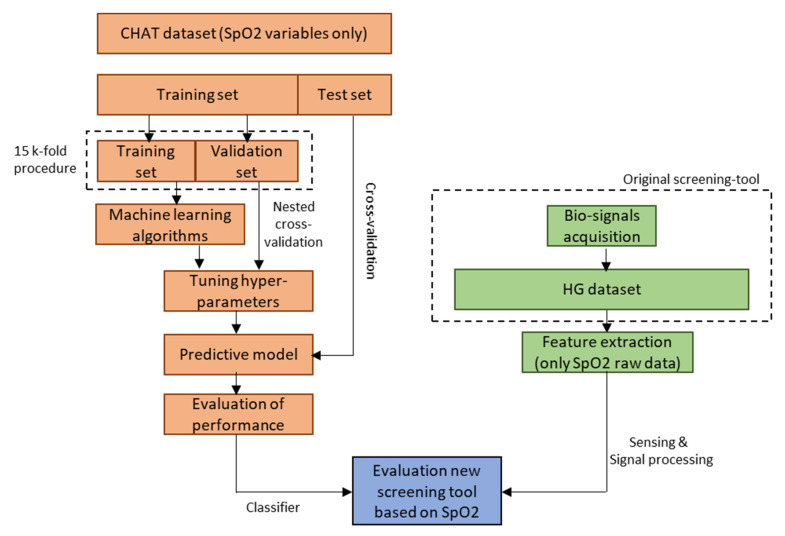
Schematic representation of the workflow followed to generate a screening tool based exclusively on pulse oximetry measurements and machine learning algorithms. This tool is specially oriented to screen asymptomatic obese pediatric population in search of subjects at risk of suffering OSA syndrome. The manuscript only focuses on the generation of this new tool, emphasizing the development of the classifier.

Finally, the area under the curve (AUC) parameter was also considered. This value is calculated after performing a receiver operating characteristic curve (ROC) analysis. It can also be assessed graphically by plotting sensitivity against 1 – specificity and determining the area under the resulting curve. A higher AUC indicates that the model has a better capability to distinguish between healthy and unhealthy subjects. Figure 1 summarizes the methodology used in this manuscript.

### 2.3. Statistical Analyses

Statistical analyses were performed on both datasets using Python [40,41,43,44]. Initially, a histogram was plotted to visualize the frequency distribution regarding the AHI values of both datasets. Afterward, a Shapiro–Wilk test was performed on the extracted features to determine if they had a normal distribution. If not, the Mann–Whitney U test was performed to determine if significant differences were present between healthy and unhealthy patients in each of the datasets. Lastly, the ROC analysis was applied to evaluate the performance of the different models by calculating the AUC parameter. A *p*-value ≤0.05 was considered to be significant.

To build the model based on the extracted features, the first step was to perform a cross-correlation matrix to determine the relationship between each pair of features as well as with AHI. The authors arbitrarily defined a strong correlation if values were greater than 0.9. In this case, dimensionality reduction was applied. This is crucial for obtaining an effective algorithm by avoiding the incorporation of repeated information into the model. This dimensionality reduction was achieved in LR using the L1 penalty term and by the mutual information measure (MI) in the SVM and AdaBoost procedures. In other words, features with lower MI were eliminated.

The selection of the best predictive model was done using a nested cross-validation method for adjusting the model parameters and estimating the error.

## 3. Results

### 3.1. Preprocessing

**Figure 2:**
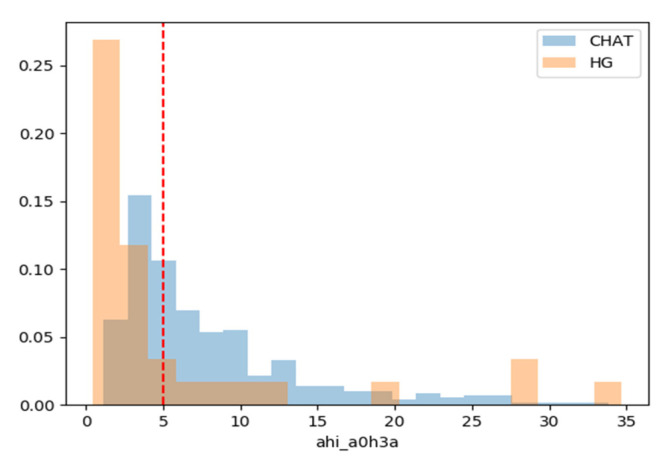
Histogram of apnea/hypopnea index of the CHAT (blue) and the Hospital General de Valencia (HG) (orange) datasets. The discontinuous red line depicts the threshold value used in the present paper, AHI = 5. Individuals with AHI ≤ 5 were considered as healthy.

Initially, the distribution of AHI for each dataset was plotted (see Figure 2). As expected, the CHAT dataset has patients with AHI ranging between 1 and 27 approximately. In contrast, the HG dataset is predominantly composed of subjects under the established threshold of 5 or with high AHI values, ≥15.

**Figure 3:**
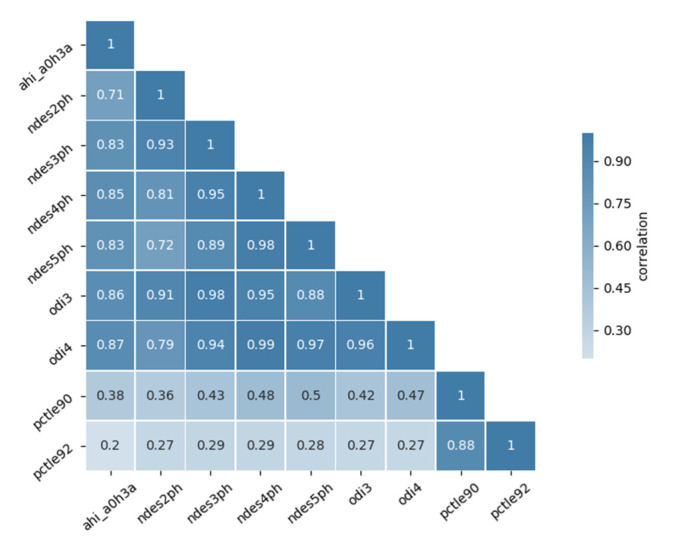
Cross-correlation matrix of the SpO2 signal features.

Afterward, Shapiro–Wilk analysis was applied to the CHAT dataset to determine if all variables had a normal distribution. As expected, this test confirmed that all variables were not normally distributed. Their *p*-values were lower than 0.05 (see Table 3). Accordingly, Mann–Whitney U analysis was performed to determine if significant differences were present between the healthy (AHI ≤ 5) and unhealthy groups (AHI > 5). Correlation analyses were performed to check the relationship between each pair of features (see Figure 3). The results reveal that strong correlations are present between odi3 and odi4 as well as ndes2ph, ndes3ph, ndes4ph and ndes5ph. Consequently, a dimensionality reduction procedure was applied to these variables before testing the three algorithms. The variables odi4 and odi3 were found to correlate the most with AHI.

**Figure 4:**
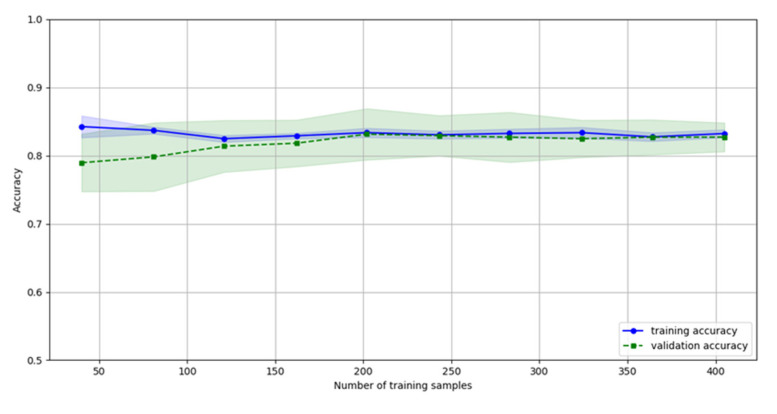
Impact of the number of samples in the overfitting of the model. The overlapping of the blue and green lines as well as the corresponding shaded areas reflect the lack of overfitting. Considering more than 120 samples mitigates overfitting.

Finally, the authors calculated the number of samples required to minimize the overfitting of the model. From Figure 4 it is deduced that the overfitting problems are negligible beyond 120 samples. The training (blue) and the testing (green) datasets yield the same or similar accuracy as the number of samples increases. Considering that the CHAT has more than 400 registries, the overfitting of the model was mitigated during the training phase.

### 3.2. Evaluating the Performance of the Tested Models

The performance of the three supervised learning classifiers was evaluated according to AUC, accuracy, recall, specificity and PPV. The results of the 15-fold process are summarized in Table 4. Focusing on the CHAT dataset, all three supervised methods yield similar AUC and accuracy. However, LR outperforms the other two regarding specificity and PPV. When applying them to the HG dataset, LR confirms its higher performance as well as higher replicability. The mean values of the different performance indicators are within the range of those yielded by more complex solutions (i.e., more than one biosignal being fed into the model and/or the use of DL algorithms to build the classifier, with the latter especially requiring domain-specific skills to design the architecture of the neural networks and to employ specific software packages) even though the accuracy is slightly lower. The fact that only a minor loss of performance is observed is very promising, especially considering the differences in age, race and SpO2 measuring devices. It is worth noting that the std value is equal to zero due to the small sample size of the HG dataset.

## 4. Discussion

The main objective of this study was to develop a screening tool, composed of a commercially available measuring device and an ML-based classifier, capable of identifying children at risk of suffering OSAS and feasible to be applied on an outpatient basis in the asymptomatic obese pediatric population. The results reveal that the proposed solution based on exclusively measuring the SpO2 signal, calculating the ODI and the NDES features and applying an LR-based classifier outperforms SVM and AdaBoost-based solutions. The performance is within the range of results achieved by methods recently developed in symptomatic adult and pediatric population and where DL-based solutions are predominant (see Table 1). Interestingly, only a minor loss of performance is observed when the LR-based classifier is applied to a second independent dataset where the participants underwent unattended NOS at home.

The prevalence of OSAS in the general pediatric population can range between 0.2–4%, but it can reach more than 50% in the obese pediatric population. Regrettably, this syndrome is not limited to a deterioration of the sleep quality of an individual, as it can cause severe health consequences (e.g., behavior regulation, compliance, aggression, impulsivity, hyperactivity, anxiety, depressed mood, emotion regulation and neurocognitive deficits) [3,4]. The current methods for diagnosing this syndrome are complex and disturbing, especially for the pediatric population, compromising the quality and reproducibility of the results. Out of all of them, full-night PSG remains the gold standard method. Besides being a very disturbing procedure, it is expensive because it requires special equipment and trained personnel to correctly detect, identify and evaluate the events from the recorded signals. Even if specialized software is available with the hardware, experts are still required to validate the results.

Alternative methods have been proposed to make these tests more patient-friendly and to reduce the workload and the diagnostic discrepancies between experts. One of the most promising due to its ease of use and low cost is the NOS, which mainly focuses on measuring the SpO2 signal, coupled with an ML- or DL-based classifier. Table 1 presents a summary of a number of these tools recently developed for symptomatic adults [16,17,18,19,20,21,22,23,24,25,26,27] and the pediatric population [28,29,30,31,32,33,34].

One major limitation of developing automated interpreting systems is choosing the most clinically meaningful biosignal. Multiple studies have tested different signal combinations to improve the performance of the classifier in detecting the OSAS severity [17,19,24,25,26,34]. Even the ECG signal, which is a commonly used parameter, has recently been questioned in terms of its continuous superior performance when compared with others [15,24]. In this context, an interesting study performed by Pathinarupothi et al. [24] showed that SpO2 outperformed ECG when a DL-based classifier was applied to each of them. The second major limitation is extracting the most distinguishable features. For example, despite the clinical relevance of ODI and of cumulative time of desaturation index (CT), not all the studies employ them [16,18,20,22,28,30,32,34]. Even when performing similar calculations, the extracted features are different or are used differently. For example, several papers used time, frequency and nonlinear domain calculations, but few or even none of them match [22,29,30,31,34]. There is also disparity when performing spectral calculations. Two recently published studies, one in adults and the other in the pediatric population, have developed tools based on images (i.e., spectrogram) [26] or based on the resulting statistic parameters [28], respectively.

Unfortunately, it is very common that these tools present a strong dataset dependency reflected by a drastic reduction of their performance when applied to a second independent dataset. Consequently, this makes it complex to compare the performance between tools and to widely implement them. DL-based solutions can overcome these limitations to an extent by using unsupervised learning algorithms to perform pattern recognition and data interpretation. However, the need for big datasets as well as domain-specific skills and software to develop and maintain these classifiers makes this approach more expensive, which limits its implementation outside clinical research where the budget of healthcare providers is already tight. In this context, the authors have focused on the development of an affordable screening tool that can be easily implementable in an obesity assessment outpatient consult to identify those asymptomatic children and adolescents who are at higher risk of suffering OSA. This is especially relevant not only for prescribing personalized physical activity strategies to fight against obesity but also for following up. This tool is composed of a commercially available device for measuring transcutaneous oxygen levels in the blood (SpO2) and an ML-based classifier for data interpretation based on the extracted features. Out of the three tested ML algorithms (i.e., LR, SVM and AB) the LR has shown better performance on the CHAT test set and after cross-validation in a second independent dataset (i.e., HG dataset). The LR outperforms the other two regarding specificity and PPV and underperforms regarding sensitivity, while all three have similar accuracy and AUC (see Table 4). This performance is promising, especially when considering that they are within the range of similar tools developed in symptomatic adults and pediatric population and using DL algorithms (e.g., neural networks). For those where an LR classifier was used [29,31], the accuracy levels are similar. However, the proposed tool has substantially higher specificity in contrast with a significantly lower sensitivity. In other words, the proposed tool is very reliable in identifying healthy patients in contrast with the others [29,31], which are better in identifying subjects at risk of suffering OSA. The findings were expected according to the purpose with which each tool was built. While [29,31] focus on determining the OSA severity of symptomatic pediatric patients, the proposed tool is reliable in identifying the healthy subjects which can immediately be prescribed with personalized physical activity-based strategies. Those which might be at risk can be confirmed or finally discarded during follow up.

Cross-validation of this tool was performed with a second independent dataset (i.e., HG). Interestingly, only a minor loss of performance was observed in the LR-based solution despite the significant differences between both datasets (e.g., age, race, the prevalence of obesity and the SpO2 measuring device). It is also relevant to point out that while the readings in CHAT were hospital-based, those in HG were home-based. Even though this loss is observed in all five indicators, specificity and PPV are the indicators that present greater reductions; this is in contrast with accuracy, recall and AUC, which only suffer a slight decrease (see Table 4). It is worth noting that the small sample size causes the prediction of all the models derived from the 15-fold process to yield identical results and therefore explains why the standard deviation is equal to 0. Out of those listed in Table 1, only the study performed by Vaquerizo-Villar et al. [32] performed cross-validation on an independent dataset. The accuracy level was 76% despite using a DL-based algorithm that was trained using power spectrum density, odi3, age and sex and targeted symptomatic children and adolescents.

Despite the need to increase the sample size of the HG dataset, these results are promising and indicate that it is worth continuing the development and optimization of the developed tool, which is expected to benefit clinicians as well as the pediatric population and their families. The former will benefit by having a tool that does not increase their workload; is easy to use and maintain; is cheap, robust and reliable; and reduces diagnosis variability between experts. The latter will benefit from a minimally disturbing tool capable of being utilized at home in an unattended manner.

## 5. Conclusions

In the recent past, alternative methods based on SpO2 measurements have been proposed to diagnose OSAS in symptomatic adults and the pediatric population. However, none of them have targeted the asymptomatic obese population even though OSAS prevalence can reach up to over 50%.

The proposed tool has shown promising results even when applied to a second independent dataset where the tests were performed on a very distinct cohort and in a home-based setting instead of a hospital-based one. Additionally, major differences were also present regarding the SpO2 measuring device. The performance yield suggests that the methodology employed can be implementable abroad. Clinicians and, in particular, the pediatric population will benefit from a tool like the one developed in this manuscript.

## Figures and Tables

**Table 1:** Summary of the performance of oxygen saturation (SpO2)-based tools to diagnose obstructive sleep apnea (OSA) syndrome.

Reference	Cohort	Type of Classifier	Sample Size	Sensitivity	Specificity	Accuracy	Year	Home-Based
[16]	A	Multivariate adaptive regression splines	793	83	54	NA	1999	N
[17]	A	Linear regression	148	91	83	89	2009	N
[18]	A	Univariate	475	96	67	87	2012	Y
[19]	A	Baggin ReTree	25	78	84	83	2012	N
[20]	A	Artificial Neural Network	93	88	100	93	2012	N
[21]	A	Univariate	996	84	86	NA	2014	Y
[22]	A	Linear discriminant analysis	302	97	50	93	2017	Y
[23]	A	Deep belief networks	33	60	92	85	2017	N
[24]	A	Long-short term memory	8	93	NA	96	2017	N
[25]	A	Convolutional neural networks	23	NA	NA	80	2018	N
[26]	A	Recurrent and convolutional neural network	15,804	NA	NA	88	2018	N
[27]	A	Common Bayesian Network	32	NA	NA	85	2017	N
[28]	P	Neural network	176	NA	NA	84.7–85.8	2015	N
[29]	P	Logistic regression	298	79.1	84.1	81.9	2017	N
[30]	P	Neural network	4191	84.0–68.7	53–94	75.2–90	2017	N
[31]	P	Logistic regression, QDA, LDA	176	NA	NA	84.3–82.7	2018	N
[32]	P	Convolutional neural network	298	NA	NA	81.3–85.3	2018	N
[33]	P	Convolutional neural network	779	40–54	98.6–99.6	74.8–95.1	2020	N
[34]	P	AdaBoost	974	91–41	22.7–98.1	78.2–85.9	2020	N

A, adult population; P, pediatric population; NA, not available; QDA, quadratic discriminant analysis; LDA, linear discriminant analysis; Y, yes; N, no.

**Table 2:** Extracted features from the raw SpO2 signal used in the Childhood Adenotonsillectomy Trial (CHAT) [6,35,36,37].

Variable	Description
**ahi_a0h3a**	Apnea/hypopnea index (AHI) ≥ 3% oxygen desaturation per hour of sleep
**odi3**	Oxygen desaturation index ≥ 3% during sleep time
**odi4**	Oxygen desaturation index ≥ 4% during sleep time
**ndes2ph**	Number of desaturations with ≥ 2% desaturation
**ndes3ph**	Number of desaturations with ≥ 3% desaturation
**ndes4ph**	Number of desaturations with ≥ 4% desaturation
**ndes5ph**	Number of desaturations with ≥ 5% desaturation
**pctle90**	Percentage of time ≤ 90% oxygen saturation
**pctle92**	Percentage of time ≤ 92% oxygen saturation

**Table 3:** Result of applying inferential statistics test on extracted features from the SpO2 raw signal.

Feature	Healthy, n = 197 (Mean ± std)	At Risk, n = 256 (Mean ± std)	Shapiro–Wilk	Mann Whitney U
			*p*-Value	*p*-Value
ndes2ph	82.91 ± 58.31	189.94 ± 107.56	< 1 × 10^−15^	< 1 × 10^−30^
ndes3ph	28.13 ± 20.45	91.67 ± 63.06	< 1 × 10^−20^	< 1 × 10^−40^
ndes4ph	10.08 ± 8.44	47.17 ± 40.32	< 1 × 10^−20^	< 1 × 10^−40^
ndes5ph	4.41 ± 4.49	26.51 ± 26.97	< 1 × 10^−25^	< 1 × 10^−40^
odi3	2.79 ± 2.08	10.53 ± 7.38	< 1 × 10^−20^	< 1 × 10^−45^
odi4	0.98 ± 0.83	5.53 ± 4.81	< 1 × 10^−25^	< 1 × 10^−45^
pctle90	0.06 ± 0.69	0.29 ± 0.51	< 1 × 10^−35^	< 1 × 10^−25^
pctle92	0.38 ± 3.49	0.81 ± 1.37	< 1 × 10^−35^	<1 × 10^−25^

ndes2ph, number of desaturations ≥ 2% per hour; ndes3ph, number of desaturations ≥ 3% per hour; ndes4ph, number of desaturations ≥ 4% per hour; ndes5ph, number of desaturations ≥ 5% per hour; odi3, oxygen desaturation index ≥ 3% during sleep time; odi4, oxygen desaturation index ≥ 4% during sleep time; pctle90, percentage of time desaturation was ≤ 90%; pctle92, percentage of time desaturation was ≤ 92%.

**Table 4:** Performance results for each algorithm in the CHAT and the HG datasets.

Dataset	Algorithm	AUC (Mean ± std)	Accuracy (Mean ± std)	Sensitivity (Mean ± std)	Specificity (Mean ± std)	PPV (Mean ± std)
CHAT	SVM	89.2 ± 7.7	82.9 ± 9.9	78.3 ± 13.5	87.4± 13.5	87.7± 12.2
	LR	90.2 ± 6.9	79.0 ± 7.2	62.0 ± 13.2	96.0 ± 5.4	94.3 ± 7.2
	AB	89.0 ± 6.7	82.1 ± 6.7	73.2 ± 11.8	90.9 ± 9.3	90.2 ± 9.8
HG	SVM	68.3 ± 4.3	66.7 ± 4.9	80.8 ± 13.6	52.5 ± 6.8	62.8 ± 4.2
	LR	85.2 ± 0.0	75.0 ± 0.0	62.5 ± 0.0	87.5 ± 0.0	83.3 ± 0.0
	AB	79.9 ± 1.3	74.6 ± 2.8	86.7 ± 3.1	62.5 ± 4.6	69.9 ± 2.7

STD, standard deviation; CHAT, Childhood Adenotonsillectomy Trial; HG, Hospital General de Valencia; SVM, support vector machine; LR, logistic regression; AB, AdaBoost; AUC, area under the curve; PPV, positive predictive value.
